# Supplementary material for: Favipiravir antiviral efficacy against SARS-CoV-2 in a hamster model
Source: Nat Commun. 2021 Mar 19;12:1735. doi: 10.1038/s41467-021-21992-w (PMC7979801; doi:10.1038/s41467-021-21992-w)
Supplement: Supplementary file 1 — Supplementary Information [file 41467_2021_21992_MOESM1_ESM.pdf]

Supplementary Information

Supplementary Figure 1: In vitro efficacy of favipiravir (T-705).

Supplementary Figure 2: Implementation of hamster model

Supplementary Figure 3: Dose-response curves

Supplementary Figure 4: Evaluation of the toxicity for animals infected and treated with high doses of favipiravir

Supplementary Figure 5: Lung histopathological changes with preemptive favipiravir therapy

Supplementary Figure 6: Clinical courses of the disease for histological analysis

Supplementary Figure 7: Plasma concentrations of favipiravir after administration of a single dose of favipiravir

Supplementary Table 1: (RT)-qPCR systems

Supplementary Table 2: Histopathological semi-quantitative lung inflammation scoring system

Supplementary Table 3: Histopathological lung inflammation semi-quantitative grading

Supplementary Table 4: Primer sequences used to produce overlapping amplicons for next generation sequencing

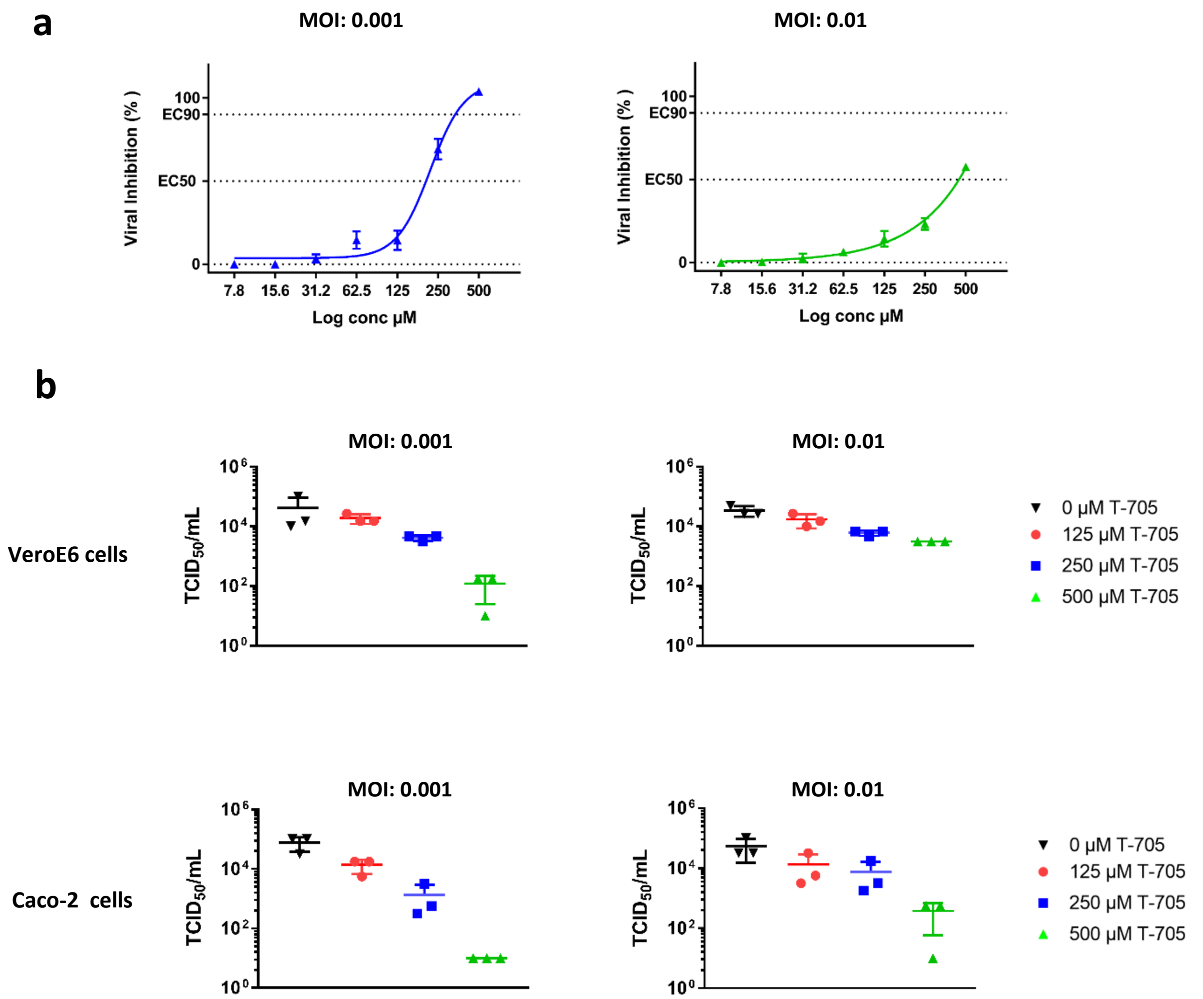

Supplementary Figure 1: In vitro efficacy of favipiravir (T-705).

a Dose-response curves of antiviral activity in VeroE6 cells (n=3 biologically independent samples). 96-well culture plates of VeroE6 cells were infected with a MOI of 0.001 or 0.01. Cells were grown three days with seven 2-fold serial dilutions of favipiravir (from 500µM to 7.8µM; in triplicate). Cytopathic effect (CPE) was measured using a cell viability assay. Percentage of viral inhibition was calculated using data from untreated cells (virus control; see methods section). **b** Infectious titer reductions (n=3 biologically independent samples). 96-well culture plates of cells (VeroE6 or Caco-2) were infected with a MOI of 0.001 or 0.01. Cells were grown three days (i) with three dilutions of favipiravir (500µM, 250µM and 125µM; in triplicate) or without drug (0µM; in triplicate). Cell supernatant media were collected to assess infectious titers using a $TCID_{50}$ assay (see methods section). Data represent mean ±SD. Source data are provided as a Source Data file.

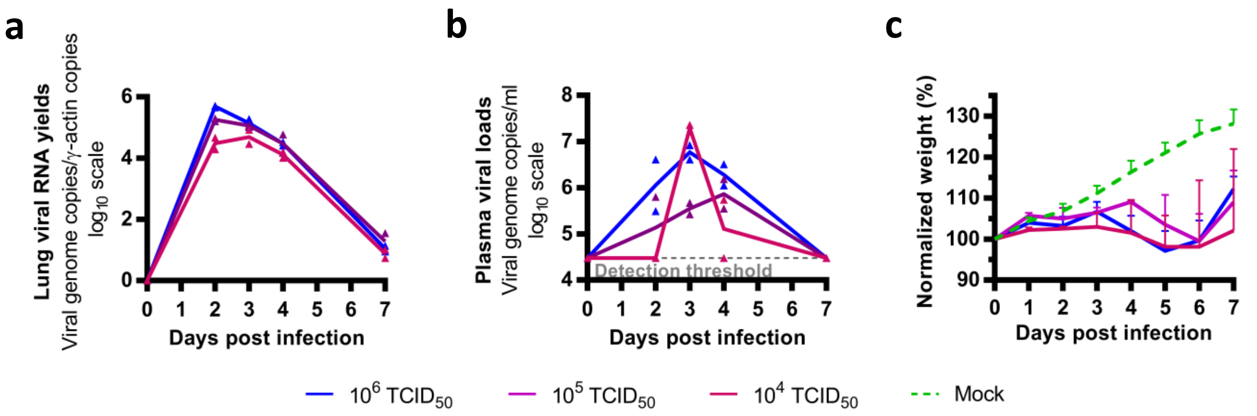

Supplementary Figure 2: Implementation of hamster model

Groups of 8 hamsters were intranasally infected with 10^6, 10^5 or 10^4 $TCID_{50}$ of virus. Viral replication was quantified using an RT-qPCR assay. **a** Lung viral RNA yields expressed in viral genome copies/copy of ɣ-actine gene for n=2 animals at days 2, 3, 4 and 7 dpi. **b** Plasma viral loads expressed in viral genome copies/mL of plasma for n=2 animals at days 2, 3, 4 and 7 dpi. **c** Clinical course of the disease for n=2 hamsters. Normalized weight at day *n* was calculated as follows: % of initial weight of the animal at day *n*. Data represent mean ±SD (details in Supplementary Data 1). Source data are provided as a Source Data file.

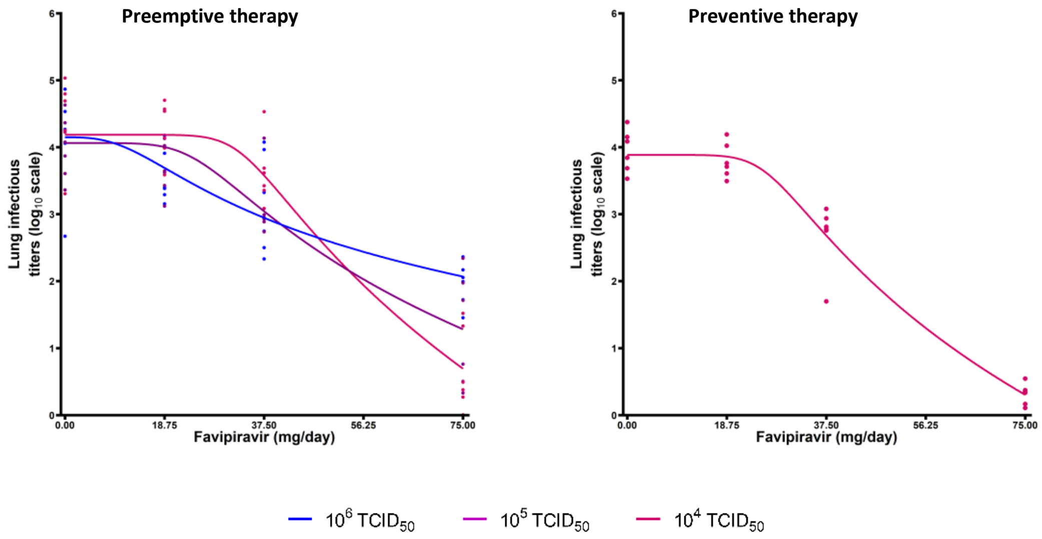

Supplementary Figure 3: Dose-response curves

Dose-response curves based on lungs infectious titers were established to determine drug 50%, 90% and 99% effective doses, for both treatment strategies (preemptive and preventive). Source data are provided as a Source Data file.

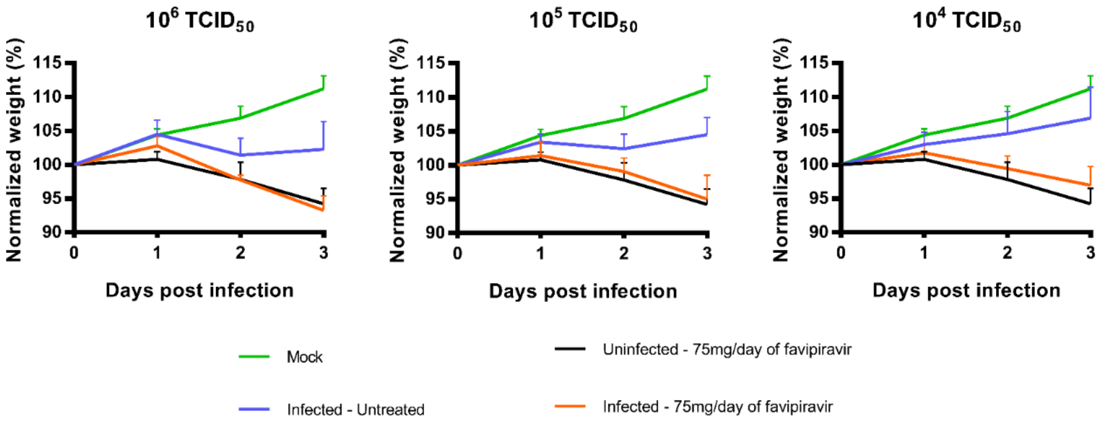

Supplementary Figure 4: Evaluation of the toxicity for animals infected and treated with high doses of favipiravir

Groups of 4 or 6 hamsters were intranasally infected with 10^6, 10^5 or 10^4 TCID$_{50}$ of virus. Clinical follow-up with animals uninfected (n=4 animals/group) or infected (n=6 animals/group) (10^6, 10^5 and 10^4 TCID$_{50}$ of virus) and untreated or treated with a dose of Favipiravir of 75mg/day TID (preemptive antiviral therapy, see figure 2). Normalized weight at day n was calculated as follows: (% of initial weight of the animal at day n)/(mean % of initial weight for mock-infected animals at day n). Data represent mean ±SD. For treated animals, no significant difference was observed between uninfected and infected animals at 1, 2 and 3 dpi (Two Way ANOVA with post-hoc Dunnett's multiple comparisons test, statistical details in Supplementary Data 6). Source data are provided as a Source Data file.

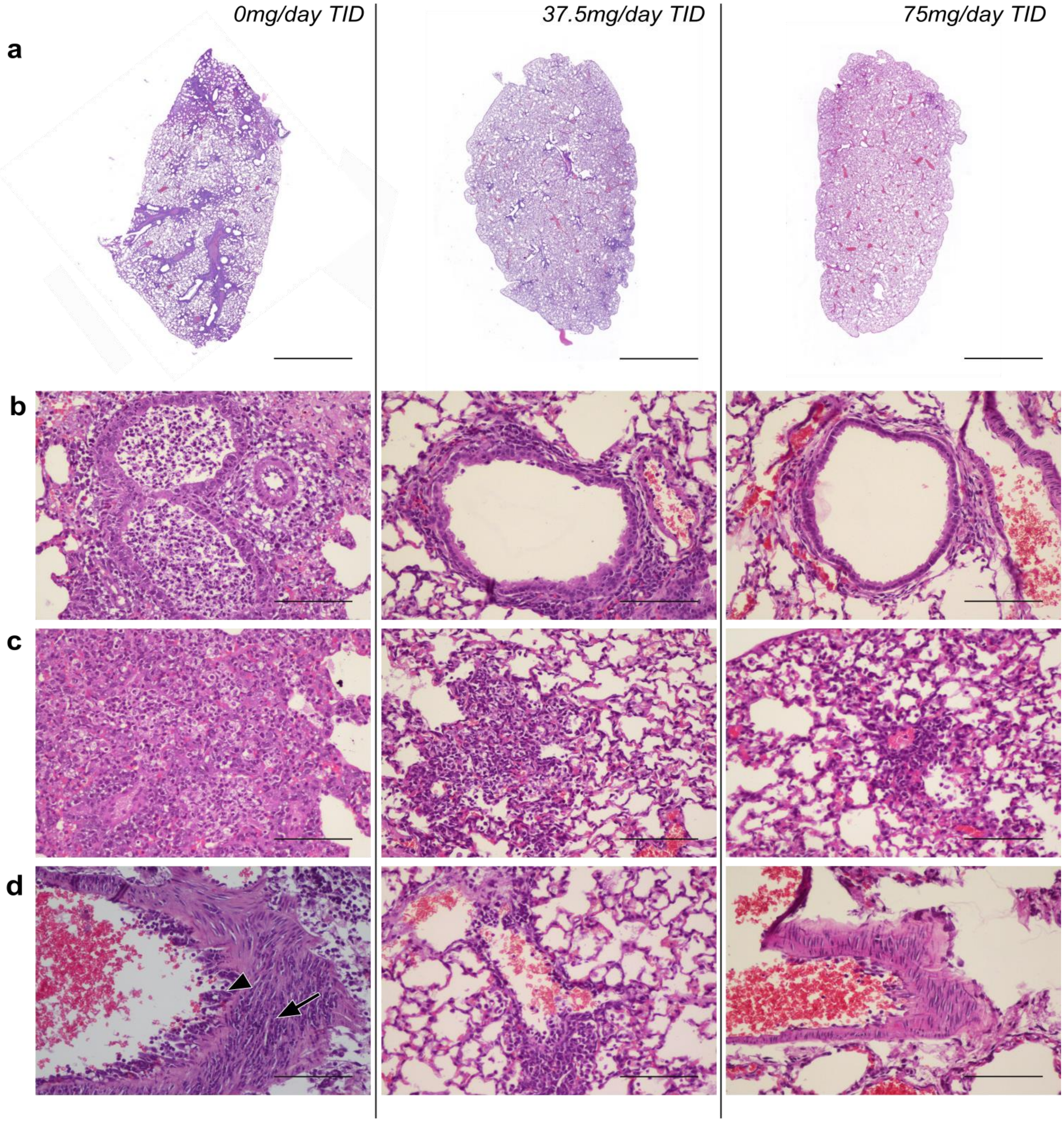

Supplementary Figure 5: Lung histopathological changes with preemptive favipiravir therapy

Groups of 4 animals were intranasally infected with 10^4 $TCID_{50}$ of virus and sacrificed at 5 dpi. At day of sacrifice, lungs were collected, fixed and embedded in paraffin. Tissue sections were stained with hematoxylin-eosin (H&E). **a** Representative images of lung tissue (left lobe) (scale bar: 2.3mm): multifocal and extensive areas of inflammation for untreated animal, multifocal but limited aeras of inflammation for 37.5mg/day treated animal and normal lung for 75mg/day treated animal (n=4 samples/group). **b** Representative images of bronchial inflammation (scale bar: 100µ): severe peribronchiolar inflammation and bronchiole filled with neutrophilic exsudates for untreated animal, mild peribonchiolar inflammation for 37.5mg/day treated animal and minimal peribronchiolar inflammation for 75mg/day treated animal. **c** Representative images of alveolar inflammation (scale bar: 100µ): severe infiltration of alveolar walls, alveoli filled with neutrophils/macrophages for untreated animal, moderate infiltration of alveolar walls, some alveoli filled with neutrophils/macrophages for 37.5mg/day treated animal and focal/mild interstitial inflammation for 75mg/day treated animal (n=4 samples/group). **d** Representative images of vessel inflammation (scale bar: 50µ): arterial marked infiltration of vascular wall with neutrophils/cell debris (arrow) and endothelial leukocytic accumulation (arrowhead) for untreated animal, venular mild leukocytes extravasation with sparse endothelial accumulation for 37.5mg/day treated animal and normal arteriol for 75mg/day treated animal (n=4 samples/group). Clinical courses of the disease are presented in Supplementary Figure 6. Statistical details are presented in Supplementary Data 7 and 8.

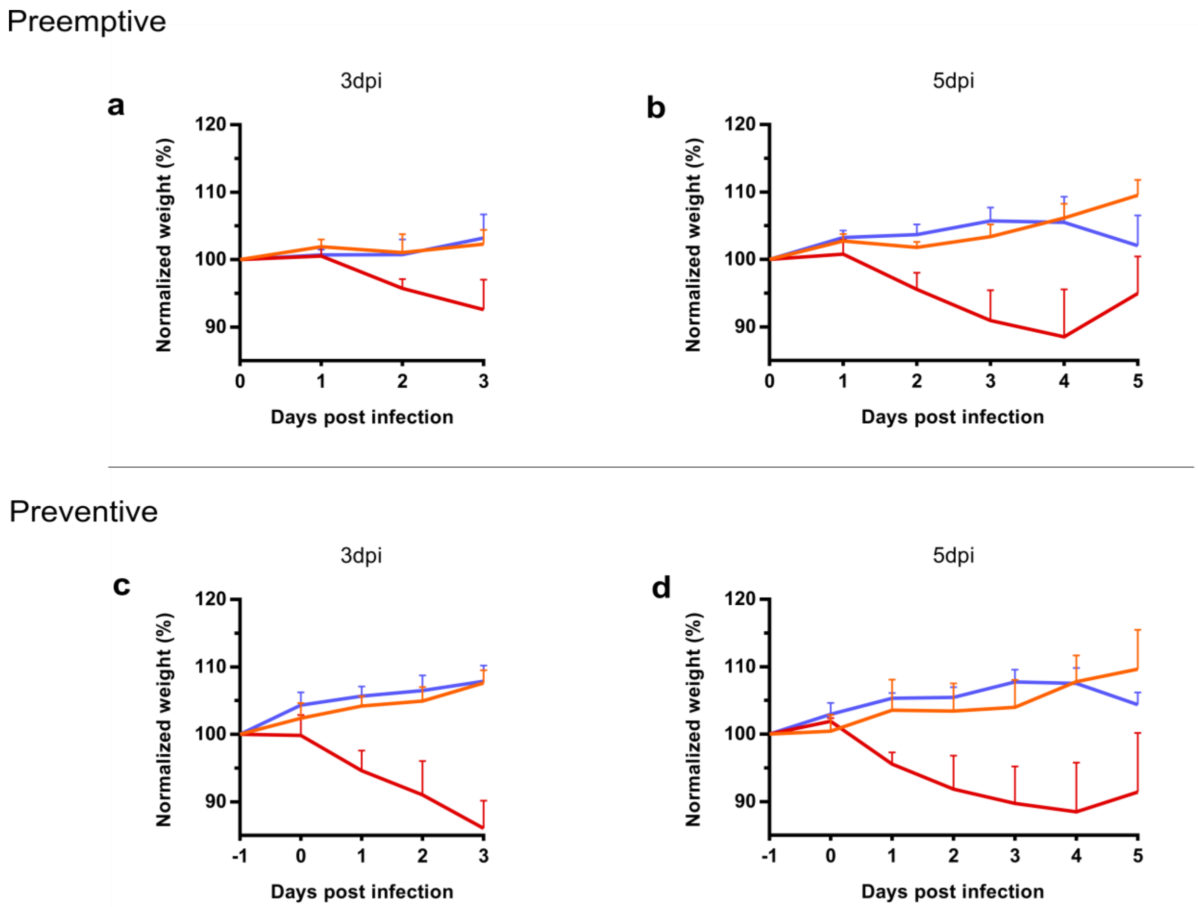

Supplementary Figure 6: Clinical courses of the disease for histological analysis

Groups of 4 hamsters were intranasally infected with 10^4 $TCID_{50}$ of virus, treated with 37.5 or 75mg/day TID following the two different treatment strategies and sacrificed at 3 and 5 dpi. Clinical course of the disease. Normalized weight at day n was calculated as follows: % of initial weight of the animal at day n (Two Way ANOVA with post-hoc Dunnett's multiple comparisons test, statistical details in Supplementary Data 7). Data represent mean ± SD. Source data are provided as a Source Data file.

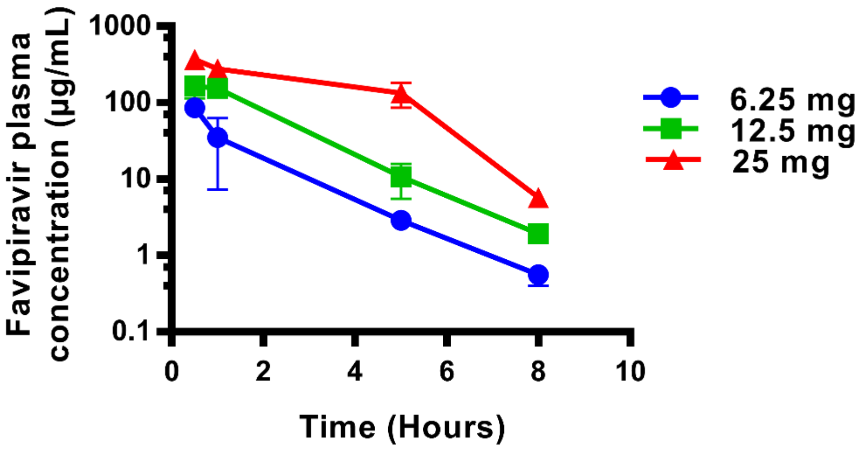

Supplementary Figure 7: Plasma concentrations of favipiravir after administration of a single dose of favipiravir

Groups of 12 uninfected animals were treated with a single dose of favipiravir (6.25, 12.5 or 25mg). Groups of 3 animals were sacrificed 30 minutes, 1 hour, 5 hours and 8 hours after the administration of the drug. Data represent mean ± SD. Source data are provided as a Source Data file.

Supplementary Table 1: (RT)-qPCR systems.

Gene Target	Primer and probes sequences	Amplicon length	Reference
Sars-CoV-2 RNA-dependent RNA polymerase	Fwd: 5'-GTGARATGGTCATGTGTGGCGG-3'<br>Rev: 5'-CARATGTTAAASACACTATTAGCATA-3'<br>Probe: 5'-FAM-CAGGTGGAACCTCATCAGGAGATGC-TAMRA-3'	99pb	Detection of 2019 novel coronavirus (2019-nCoV) by real-time RT-PCR (Corman et al.)
Syrian hamster γ-actin	Fwd: 5'-ACAGAGAGAAGATGACGCAGATAATG-3'<br>Rev: 5'-GCCTGAATGGCCACGTACA-3'<br>Probe: 5'-FAM-TTGAAACCTTCAACACCCCAGCC-TAMRA-3'	70pb	Duplex real-time reverse transcriptase PCR to determine cytokine mRNA expression in a hamster model of New World cutaneous leishmaniasis (Espitita et al.)
Bacteriophage MS2	Fwd: 5'-CTCTGAGAGCGGCTCTATTGGT-3'<br>Rev: 5'-GTTCCCTACAACGAGCCTAAATTC-3'<br>Probe: 5'-VIC-TCAGACACGCGGTCCGCTATAACGA-TAMRA-3'	100pb	RNA and DNA Bacteriophages as Molecular Diagnosis Controls in Clinical Virology: A Comprehensive Study of More than 45,000 Routine PCR Tests (Ninove et al.)

Supplementary Table 2: Histopathological semi-quantitative lung inflammation scoring system

Lesion	Description	Intensity	Score
**Interstitial pneumonia**	1 o 2 foci with 10-20 cells or small area with two-fold thickening of alveolar septa	Mild	1
	3 to 5 foci with 10-30 cells or widespread areas with two-fold thickening of alveolar septa	Moderate	2
	5 foci of 10-50 cells or widespread areas with two-fold or three-fold thickening of alveolar septa throughout the lung	Marked	3
	5 foci of 10-100 cells or widespread areas with three to fourfold-thickened alveolar septa throughout the lung	Severe	4
**Bronchitis**	1 or 2 bronchi section(s) filled with rare necrotic/inflammatory cells or partially surrounded by scarce inflammatory cells	Mild	1
	3 to 5 bronchi filled with necrotic/inflammatory cells or partially surrounded by a few inflammatory cells	Moderate	2
	6 to 10 bronchi filled with necrotic/inflammatory cells, partially or sub-completely surrounded by inflammatory cells	Marked	3
	Numerous bronchi filled with inflammatory or cellular debris or completely surrounded by numerous inflammatory cells	Severe	4
**Endothelitis, vasculitis**	Absent	-	0
	Present	-	1
**Hemorrhagic necrosis**	Absent	-	0
	Focal to multifocal	Mild to moderate	1
	Coalescing to extensive necrosis	Severe	2

Supplementary Table 3: Histopathological lung inflammation semi-quantitative grading

Cumulative score	Grade	Bronchointerstitial pneumonia
0	0	Normal
1-3	1	Mild
4-5	2	Moderate
6-8	3	Marked
9-10	4	Severe

Supplementary Table 4: Primer sequences used to produce overlapping amplicons for next generation sequencing.

Name	Primer Sequence	Start	End	Tm	GC%
1Forward	ACCAACCAACTTTCGATCTCTTGT	31	54	60.69	41.67
1Reverse	TTTCGAGCAACATAAGCCCGTT	2621	2642	61.13	45.45
2Forward	AACAACCTACTAGTGAAGCTGTTGA	2565	25989	60.16	40.00
2Reverse	TTGACATGTCCACAACTTGCGT	5006	5027	61.26	45.45
3Forward	CTTCTTTCTTTGAGAGAAGTGAGGACT	4940	4966	60.69	40.74
3Reverse	TGCCAAAAACCACTCTGCAACT	7234	7255	61.47	45.45
4Forward	GTGGTTTAGATTCTTTAGACACCTATCCT	7143	7171	60.59	37.93
4Reverse	AGGTGTGAACATAACCATCCACTG	9644	9667	60.81	45.83
5Forward	ACTCATTCTTACCTGGTGTTTATTCTGT	9558	9585	60.69	35.71
5Reverse	CTGGACACATTGAGCCCACAAT	11923	11944	61.14	50.00
6Forward	TGCACATCAGTAGTCTTACTCTCAGT	11864	11889	61.25	42.31
6Reverse	TGTGACTCTGCAGTTAAAGCCC	14186	14207	60.81	50.00
7Forward	AGACGGTGACATGGTACCACAT	13758	13779	61.41	50.00
7Reverse	ACACGTTGTATGTTTGCGAGCA	15354	15375	61.63	45.45
8Forward	TGATTGTTACGATGGTGGCTGT	14880	14901	60.29	45.45
8Reverse	GTGCAGGTAATTGAGCAGGGTC	17437	17458	61.52	54.55
9Forward	TGATTTGAGTGTTGTCAATGCCAG	17382	17405	60.26	41.67
9Reverse	ATTAGCAGCAATGTCCACACCC	19845	19886	61.21	50.00
10Forward	AATGTAGCATTTGAGCTTTGGGC	19774	19796	60.37	43.48
10Reverse	ACCAGCTGTCCAACCTGAAGAA	22324	22345	61.82	50.00
11Forward	ACATCACTAGGTTTCAAACTTTACTTGC	22263	22290	60.68	35.71
11Reverse	ATGAGGTGCTGACTGAGGGAAG	24715	24736	61.74	54.55
12Forward	GTCAGAGTGTGTACTTGGACAATCA	24649	24673	60.74	44.00
12Reverse	ACTGCTACTGGAATGGTCTGTGT	27142	27164	61.58	47.83
13Forward	GGTGACTCAGGTTTTGCTGCAT	27087	27108	61.65	50.00
13Reverse	CGTAAACGGAAAAGCGAAAACGT	29571	59593	61.08	43.48

The primers used come from a larger series of primer intended initially for carrying out multiplexed PCR obtained on the site http://primal.zibraproject.org/, acording to Quick J et al.

Multiplex PCR method for MinION and Illumina sequencing of Zika and other virus genomes directly from clinical samples. Nat Protoc. 2017 Jun;12(6):1261-1276.
