# Supplementary material for: Favipiravir antiviral efficacy against SARS-CoV-2 in a hamster model
Source: Nat Commun. 2021 Mar 19;12:1735. doi: 10.1038/s41467-021-21992-w (PMC7979801; doi:10.1038/s41467-021-21992-w)
Supplement: Supplementary file 3 — Description of Additional Supplementary Data [file 41467_2021_21992_MOESM3_ESM.docx]

**Description of Additional Supplementary Data**

**File name: Supplementary Data 1**

Description: Implementation of hamster model. Gross weights and viral RNA yields in organs.

**File name: Supplementary Data 2**

Description: Individual data from in vivo experiments. Gross body weights, lung infectious titers, lung viral RNA yields, lung infectivities and plasmatic viral RNA yields.

**File name: Supplementary Data 3**

Description: Statistical analysis of in vivo experiments.

**File name: Supplementary Data 4**

Description: Normality test analysis of in vivo experiment variables.

**File name: Supplementary Data 5**

Description: Statistical analysis of clinical monitoring.

**File name: Supplementary Data 6**

Description: Statistical analysis of in vivo experiments presented in Figure S4.

**File name: Supplementary Data 7**

Description: Individual data and statistical analysis of histopathological experiments.

**File name: Supplementary Data 8**

Description: Statistical analysis of histopathologic changes in hamsters lungs.

**File name: Supplementary Data 9**

Description: Individual data of favipiravir pharmacokinetics.

**File name: Supplementary Data 10**

Description: Individual data for analysis of mutagenic effect of favipiravir.

**File name: Supplementary Data 11**

Description: Statistical analysis of mutagenic effect of favipiravir.

**File name: Supplementary Data 12**

Description: Normality test analysis of favipiravir's mutagenic effect variables.

**File name: Supplementary Data 13**

Description: Shared mutations detected in lung clarified homogenates.
