# Supplementary material for: Favipiravir antiviral efficacy against SARS-CoV-2 in a hamster model
Source: Nat Commun. 2021 Mar 19;12:1735. doi: 10.1038/s41467-021-21992-w (PMC7979801; doi:10.1038/s41467-021-21992-w)
Supplement: Supplementary file 17 — Reporting Summary [file 41467_2021_21992_MOESM17_ESM.pdf]

nature research

Corresponding author(s): Antoine Nougairède

Last updated by author(s): Dec 1, 2021

Reporting Summary

Nature Research wishes to improve the reproducibility of the work that we publish. This form provides structure for consistency and transparency in reporting. For further information on Nature Research policies, see our Editorial Policies and the Editorial Policy Checklist.

Statistics

For all statistical analyses, confirm that the following items are present in the figure legend, table legend, main text, or Methods section.

n/a	Confirmed	
☐	☒	The exact sample size (n) for each experimental group/condition, given as a discrete number and unit of measurement
☐	☒	A statement on whether measurements were taken from distinct samples or whether the same sample was measured repeatedly
☐	☒	The statistical test(s) used AND whether they are one- or two-sided<br>*Only common tests should be described solely by name; describe more complex techniques in the Methods section.*
☐	☒	A description of all covariates tested
☐	☒	A description of any assumptions or corrections, such as tests of normality and adjustment for multiple comparisons
☐	☒	A full description of the statistical parameters including central tendency (e.g. means) or other basic estimates (e.g. regression coefficient) AND variation (e.g. standard deviation) or associated estimates of uncertainty (e.g. confidence intervals)
☐	☒	For null hypothesis testing, the test statistic (e.g. F, t, r) with confidence intervals, effect sizes, degrees of freedom and P value noted<br>*Give P values as exact values whenever suitable.*
☒	☐	For Bayesian analysis, information on the choice of priors and Markov chain Monte Carlo settings
☒	☐	For hierarchical and complex designs, identification of the appropriate level for tests and full reporting of outcomes
☒	☐	Estimates of effect sizes (e.g. Cohen's d, Pearson's r), indicating how they were calculated

Our web collection on statistics for biologists contains articles on many of the points above.

Software and code

Policy information about availability of computer code

Data collection	QuantStudio 12K Flex Software v1.2.3. MassLynx Mass Spectrometry Software 4.1.
Data analysis	R statistical software (http://www.R-project.org). Graphpad Prism 7. CLC genomics workbench software v.20 (Qiagen). software Pkanalix2019R2. Excel 2016.

For manuscripts utilizing custom algorithms or software that are central to the research but not yet described in published literature, software must be made available to editors and reviewers. We strongly encourage code deposition in a community repository (e.g. GitHub). See the Nature Research guidelines for submitting code & software for further information.

Data

Policy information about availability of data

All manuscripts must include a data availability statement. This statement should provide the following information, where applicable:

- Accession codes, unique identifiers, or web links for publicly available datasets
- A list of figures that have associated raw data
- A description of any restrictions on data availability

Raw sequence reads of the virus genome analysed in this study have been deposited in the BioProject data bank (PRJNA648821). Authors can confirm that all other relevant data are included in the paper and/or its supplementary information files.

Field-specific reporting

Please select the one below that is the best fit for your research. If you are not sure, read the appropriate sections before making your selection.

[x] Life sciences [ ] Behavioural & social sciences [ ] Ecological, evolutionary & environmental sciences

For a reference copy of the document with all sections, see nature.com/documents/nr-reporting-summary-flat.pdf

Life sciences study design

All studies must disclose on these points even when the disclosure is negative.

Sample size	No sample-size calculation was performed prior the experience. This was done due to the complete lack of previous data concerning Sars-CoV-2 in vivo experiments at that time. However, sample size were determined based on previously published experiments with favipiravir against several viruses. Between 3 and 10 hamsters are generally used in these studies. Therefore we have choosen to work 6 hamsters, while also taking into consideration the three Rs (Replacement, Reduction, Refinement) principles for animal experimentation.<br><br>References:<br>Dawes BE, Kalveram B, Ikegami T, Juelich T, Smith JK, Zhang L, Park A, Lee B, Komeno T, Furuta Y, Freiberg AN. Favipiravir (T-705) protects against Nipah virus infection in the hamster model. Sci Rep. 2018 May 15;8(1):7604. doi: 10.1038/s41598-018-25780-3. PMID: 29765101; PMCID: PMC5954062.<br>Gowen BB, Westover JB, Miao J, Van Wettere AJ, Rigas JD, Hickerson BT, Jung KH, Li R, Conrad BL, Nielson S, Furuta Y, Wang Z. Modeling Severe Fever with Thrombocytopenia Syndrome Virus Infection in Golden Syrian Hamsters: Importance of STAT2 in Preventing Disease and Effective Treatment with Favipiravir. J Virol. 2017 Jan 18;91(3):e01942-16. doi: 10.1128/JVI.01942-16. PMID: 27881648; PMCID: PMC5244333.<br>Jochmans D, van Nieuwkoop S, Smits SL, Neyts J, Fouchier RA, van den Hoogen BG. Antiviral Activity of Favipiravir (T-705) against a Broad Range of Paramyxoviruses In Vitro and against Human Metapneumovirus in Hamsters. Antimicrob Agents Chemother. 2016 Jul 22;60(8):4620-9. doi: 10.1128/AAC.00709-16. PMID: 27185803; PMCID: PMC4958190.<br>Westover JB, Sefing EJ, Bailey KW, Van Wettere AJ, Jung KH, Dagley A, Wandersee L, Downs B, Smee DF, Furuta Y, Bray M, Gowen BB. Low-dose ribavirin potentiates the antiviral activity of favipiravir against hemorrhagic fever viruses. Antiviral Res. 2016 Feb;126:62-8. doi: 10.1016/j.antiviral.2015.12.006. Epub 2015 Dec 19. PMID: 26711718; PMCID: PMC4724333.<br>Safronetz D, Falzarano D, Scott DP, Furuta Y, Feldmann H, Gowen BB. Antiviral efficacy of favipiravir against two prominent etiological agents of hantavirus pulmonary syndrome. Antimicrob Agents Chemother. 2013 Oct;57(10):4673-80. doi: 10.1128/AAC.00886-13. Epub 2013 Jul 15. PMID: 23856782; PMCID: PMC3811478.<br>Julander JG, Shafer K, Smee DF, Morrey JD, Furuta Y. Activity of T-705 in a hamster model of yellow fever virus infection in comparison with that of a chemically related compound, T-1106. Antimicrob Agents Chemother. 2009 Jan;53(1):202-9. doi: 10.1128/AAC.01074-08. Epub 2008 Oct 27. PMID: 18955536; PMCID: PMC2612161.
Data exclusions	No data were excluded.
Replication	Data presented in Figure 1 come from three independent experiments. Data presented in Figure 2 come from one independent experiment. Data presented in Figure 3 come from one independent experiment. Data presented in Figure 4 come from one independent experiment. Overall, as demonstrated by our results, we found consistency between all these independent experiments.
Randomization	Hamsters were placed randomly in their cage and cages were randomly assigned to a treatment. For in vitro study, randomization is irrelevant.
Blinding	Blindind was impossible in our study since it was the same investigators who prepared the drug solution, administrated the drug each days, monitored the hamsters each days, sacrificed the hamsters, collected the organs and performed all the data analysis. At each step, it was essential to identify each group. Blinding was used only for the histopathological study (blinded analysis of lung samples).

Reporting for specific materials, systems and methods

We require information from authors about some types of materials, experimental systems and methods used in many studies. Here, indicate whether each material, system or method listed is relevant to your study. If you are not sure if a list item applies to your research, read the appropriate section before selecting a response.

Materials & experimental systems

n/a	Involved in the study	
x		Antibodies
	x	Eukaryotic cell lines
x		Palaeontology and archaeology
	x	Animals and other organisms
x		Human research participants
x		Clinical data
x		Dual use research of concern

Methods

n/a	Involved in the study	
x		ChIP-seq
x		Flow cytometry
x		MRI-based neuroimaging

Eukaryotic cell lines

Policy information about cell lines

Cell line source(s)	VeroE6 cells (ATCC CRL-1586). Caco-2 cells (ATCC HTB-37)
Authentication	The cell lines have not been authenticated since it was purchased from ATCC.
Mycoplasma contamination	Cell lines were tested negative for mycoplasma contamination.
Commonly misidentified lines (See ICLAC register)	No commonly misidentified lines were used.

Animals and other organisms

Policy information about studies involving animals; ARRIVE guidelines recommended for reporting animal research

Laboratory animals	Three-week-old female Syrian hamsters were provided by Janvier Labs.
Wild animals	The study did not involve wild animals.
Field-collected samples	The study did not involve sample collected from the field.
Ethics oversight	Experiments were approved by the local ethical committee (C2EA—14) and the French 'Ministère de l'Enseignement Supérieur, de la Recherche et de l'Innovation' (APAFIS#23975) and performed in accordance with the French national guidelines and the European legislation covering the use of animals for scientific purposes.

Note that full information on the approval of the study protocol must also be provided in the manuscript.
